# 4463 An Intervention Mapping Approach to Develop Interventions to Improve Access to Kidney Transplant

**DOI:** 10.1017/cts.2020.254

**Published:** 2020-07-29

**Authors:** Hannah D’Cunha, Melissa Partin, Warren McKinney, Marilyn Bruin, Allyson Hart

**Affiliations:** 1 University of Minnesota CTSI

## Abstract

OBJECTIVES/GOALS: Kidney transplant is superior to dialysis for the treatment of end-stage kidney disease, but accessing transplant requires high patient engagement. We sought to develop a group counseling intervention with patients and their social support members using an evidence-based, stakeholder-engaged approach. METHODS/STUDY POPULATION: We employed an Intervention Mapping approach to incorporate qualitative data from stakeholders on barriers to accessing kidney transplant. Data were collected from 13 focus groups of African American (AA) and white adult kidney transplant candidates and their social support networks in Minnesota and Georgia. We completed this process through (1) qualitative data collection, (2) utilizing data and intervention mapping methods to develop a conceptual framework to describe associations between behavioral determinants and desired outcomes, and (3) using these products to identify evidence-based approaches to modify behavioral determinants through a theory based intervention. RESULTS/ANTICIPATED RESULTS: Participants describe experiences of overwhelm, isolation, helplessness, and difficulty communicating. In addition, AA participants expressed distrust in the medical system. We systematically incorporated these themes into a conceptual model of behavior change that identifies determinants of necessary actions to obtain transplant, including knowledge, self-efficacy, reduced decisional conflict, and perception of social support. Evidence-based methods to modify these determinants, such as modeling, goal-setting, and mobilizing social support, were incorporated into the design of a group education and counseling intervention with an individualized risk calculator. DISCUSSION/SIGNIFICANCE OF IMPACT: Intervention mapping allows for behavior change theories to be incorporated into counseling sessions with patients and their social support networks. This approach translates qualitative data into an evidence-based intervention which will be piloted in a randomized controlled trial (RCT) to determine feasibility for a larger RCT.

